# Bone Aging, Cellular Senescence, and Osteoporosis

**DOI:** 10.1002/jbm4.10488

**Published:** 2021-04-02

**Authors:** Robert J Pignolo, Susan F Law, Abhishek Chandra

**Affiliations:** ^1^ Department of Medicine Mayo Clinic Rochester MN USA; ^2^ Department of Physiology and Biomedical Engineering Mayo Clinic Rochester MN USA

**Keywords:** AGING, BONE, CELLULAR SENESCENCE, OSTEOPOROSIS, SENOLYTICS

## Abstract

Changes in aging bone that lead to osteoporosis are mediated at multiple levels, including hormonal alterations, skeletal unloading, and accumulation of senescent cells. This pathological interplay is superimposed upon medical conditions, potentially bone‐wasting medications, modifiable and unmodifiable personal risk factors, and genetic predisposition that accelerate bone loss with aging. In this study, the focus is on bone hemostasis and its dysregulation with aging. The major physiological changes with aging in bone and the role of cellular senescence in contributing to age‐related osteoporosis are summarized. The aspects of bone aging are reviewed including remodeling deficits, uncoupling phenomena, inducers of cellular senescence related to bone aging, roles of the senescence‐associated secretory phenotype, radiation‐induced bone loss as a model for bone aging, and the accumulation of senescent cells in the bone microenvironment as a predominant mechanism for age‐related osteoporosis. The study also addresses the rationale and potential for therapeutic interventions based on the clearance of senescent cells or suppression of the senescence‐associated secretory phenotype. © 2021 The Authors. *JBMR Plus* published by Wiley Periodicals LLC on behalf of American Society for Bone and Mineral Research.

## Introduction

As part of the skeleton, bone tissue functions to support locomotion, hematopoiesis, glucose metabolism, interactions with the renal and reproductive systems, and reservoirs for phosphorus and calcium, as well as protection for internal organs.^(^ ^1^ ^)^ Bone is made up of extracellular matrix proteins, inorganic mineral in the form of hydroxyapatite, and many resident cell types. The formation of bone during normal development and accrual after physiological growth plate closure mainly occurs in the first two decades of life in healthy individuals, after which BMD plateaus and is followed by age‐related bone loss.^(^ ^2^ ^)^ Osteoporosis in women typically occurs during the postmenopausal period, and in both women and men caused by age‐related changes.^(^ ^3^, ^4^ ^)^

## Bone Remodeling and Aging

The deposition of bone matrix, its mineralization, and remodeling is regulated by several cell types. One such cell type, the osteoclast, is a large multinucleate cell that absorbs bone and differentiates from bone marrow monocyte/macrophage precursors. Osteoclast differentiation is promoted by osteoblasts, osteocytes, and activated T lymphocytes^(^ ^5^, ^6^, ^7^ ^)^ by the secretion of receptor activator of nuclear factor kappa‐B (RANK) ligand (RANKL), which binds to the RANK receptor on the osteoclast surface.^(^ ^8^, ^9^ ^)^ RANK receptor expression is increased by the binding of macrophage colony‐stimulating factor (M‐CSF), secreted by osteoblasts and bone marrow stromal cells,^(^ ^10^ ^)^ to the colony‐stimulating factor‐1 receptor also known as c‐FMS on the osteoclast surface. Mature osteoclasts absorb bone by secreting acid and proteolytic enzymes that dissolve the bone matrix. Osteoclast differentiation and activity can be inhibited by osteoprotegerin (OPG), a decoy receptor that binds RANKL and is secreted by osteoblasts, osteocytes, B lymphocytes, and the liver.^(^ ^6^, ^11^ ^)^

In contrast, osteoblasts promote new bone formation. Osteoblasts differentiate from bone mesenchymal stem cells (BMSCs) in response to WNT signaling. WNT signaling accomplishes this by stabilizing β‐catenin which directs the transcription of genes involved in osteoblast differentiation like Runt‐related transcription factor 2 (Runx2) and osterix (Osx)^(^ ^12^ ^)^ while adipogenesis and CCAAT‐enhancer binding protein α are inhibited.^(^ ^13^ ^)^ WNT signaling in osteoblasts can be inhibited by sclerostin and Dickkopf‐1 (DKK1).^(^ ^14^ ^)^ Mature osteoblasts secrete osteocalcin, alkaline phosphatase, and type I collagen, the predominant component of the matrix,^(^ ^15^ ^)^ after which mineralization occurs with calcium‐phosphate in the form of hydroxyapatite.

Osteocytes are terminally differentiated osteoblasts that are found embedded in the mineralized matrix, are long‐lived, and are important for sensing mechanical load.^(^ ^16^ ^)^ An adult skeleton has an estimated 42 × 10^9^ osteocytes occupying 215 m^2^ of lacunar‐canalicular surface area^(^ ^17^ ^)^ allowing osteocytes to release a significant amount of calcium and signaling molecules as a way to maintain mineral homeostasis and direct bone remodeling. Osteocytes secrete many signaling molecules that both positively and negatively affect bone remodeling.^(^ ^18^ ^)^ They are a major source of RANKL promoting osteoclastogenesis,^(^ ^19^ ^)^ as well as sclerostin and DKK1, which antagonize WNT signaling and inhibit osteoblast bone formation.^(^ ^14^ ^)^ The bone marrow holds many cell types such as those of hematopoietic lineages including myeloid (osteoclast precursor), lymphoid, and erythroid cells, as well as marrow stromal cells and BMSCs that can differentiate into osteoblasts, adipocytes, and chondrocytes.^(^ ^20^ ^)^ Bone is not a static structure but is metabolically active in maintaining mineral homeostasis; it is constantly remodeling in response to many influences, including mechanical loading, and hormonal and immunologic pressures.

Normal bone remodeling occurs throughout the skeleton and is a balance between resorption of existing bone (old, weak, or damaged) and new bone deposition.^(^ ^21^ ^)^ It occurs in a sequential manner: (i) activation—when osteoclasts are recruited to damaged or otherwise incompetent bone, (ii) resorption—when mature osteoclasts resorb bone, (iii) reversal—when osteoclasts die and osteoblast progenitors are recruited, and (iv) formation—when mature osteoblasts deposit new bone.^(^ ^22^, ^23^ ^)^ Many secreted factors from osteoclasts and osteoblasts have been identified as important for bone remodeling.^(^ ^18^, ^24^ ^)^ In addition, membrane‐bound mediators such as Ephrin family proteins are important in signaling cascades activated by direct cell–cell contact between osteoclasts and osteoblasts.^(^ ^18^ ^)^ Matrix‐associated proteins also play a role in bone remodeling, linking bone resorption and formation. Transforming growth factor β1 (TGF‐β1) and insulin‐like growth factor type I (IGF‐1) reside in the bone matrix in their inactive form and upon osteoclast resorption are activated to promote mesenchymal cell differentiation to form mature osteoblasts.^(^ ^25^, ^26^, ^27^, ^28^ ^)^ Orchestration of bone remodeling occurs at the direction and interplay of these cells and the secreted factors present. In contrast, there are other situations in which bone resorption and formation are not sequential and this is termed bone modeling. Bone modeling occurs during growth and periosteal expansion when bone is deposited, and can be lost in the case of some pharmacologic interventions and pathological skeletal disorders like inflammatory bone loss.^(^ ^29^ ^)^

Recently, microRNAs (miRNAs) have been shown to play an important role in bone remodeling. miRNAs are evolutionarily conserved noncoding small RNAs that regulate gene expression posttranscriptionally by binding to the 3′‐untranslated region of mRNA to block translation or promote degradation.^(^ ^30^ ^)^ Regulation of biological processes by miRNA is complicated as each miRNA—of the thousands that have been identified—can target many transcripts and functionally overlap with other miRNAs.^(^ ^31^ ^)^ To add to the complexity, miRNAs work not only inside the cell, but can be transported via exosomes to surrounding cells^(^ ^32^ ^)^ and at a distance by the bloodstream and other bodily fluids.^(^ ^33^ ^)^ In an example of miRNA control, a study in osteoblasts identified 11 miRNAs that directly inhibit Runx2 protein production, although at different levels and with most able to inhibit osteoblast differentiation in a reversible manner.^(^ ^34^ ^)^ Osteoclast formation has likewise been shown to be influenced by miRNAs. miR‐26a is upregulated late in osteoclastogenesis, with ectopic expression attenuating osteoclast and actin‐ring formation, as well as bone resorption by inhibiting the expression of connective tissue growth factor (CTGF)/CCN family 2, which could be prevented with the addition of recombinant CTGF.^(^ ^35^ ^)^ Similarly, osteocytes are regulated by miRNAs, and their miRNA‐containing exosomes can influence the functioning of bone cells.^(^ ^30^ ^)^ Interestingly, in an example of cell‐to‐cell communication, miR‐214‐3p can be secreted by osteoclasts and then taken up by osteoblasts. The miR‐214‐3p can then inhibit osteoblast activity in vitro and bone formation in vivo and is elevated in the serum of elderly women who show reduced bone formation with fractures.^(^ ^36^ ^)^ The study of miRNAs is progressing rapidly, but given the complexity of possible regulatory networks much remains to be uncovered.

In humans, trabecular bone loss begins in the third decade of life in both men and women and substantially increases in the perimenopause period.^(^ ^37^, ^38^ ^)^ This process is directly affected by decreases in estrogen and testosterone levels, leading to bone loss and potentially osteoporosis. ^(^ ^37^ ^)^ Other hormones also affect bone homeostasis, including PTH and corticosteroids.^(^ ^39^, ^40^ ^)^ For example, secondary hyperparathyroidism caused by low vitamin D levels and decreased renal calcium absorption with aging is a common phenomenon.^(^ ^41^ ^)^ The changes in trabecular architecture include decreases in trabecular number, which is greater in women than in men,^(^ ^38^ ^)^ a decreased trabecular thickness that is greater in men than women,^(^ ^42^ ^)^ and a loss of connectivity. After menopause in women and sex steroid deficiency in men, cortical bone loss with increases in porosity has been documented.^(^ ^43^ ^)^ Structurally, aging also affects the lacunar‐canalicular system with human histomorphometric studies showing a decrease in lacunar density with a loss ranging from 15% to 30%.^(^ ^44^, ^45^ ^)^ The shrinkage of the canalicular network with age^(^ ^46^, ^47^ ^)^ affects intercellular communication and responses to skeletal loading and exercise on bone formation. Connexin‐43 is a protein that maintains the canalicular network; the loss of connexin‐43 with aging promotes osteocyte cell death, the appearance of empty lacunae, the recruitment of osteoclasts, and alterations in bone material properties.^(^ ^48^ ^)^ Furthermore, loss of osteocyte lacunar density in human cortical bone triggers the accumulation of microcracks, a sign of bone deterioration contributing to osteoporosis.^(^ ^49^ ^)^

In the bone marrow cavity, marrow adipose tissue increases with age causing the development of a yellow fatty marrow.^(^ ^50^ ^)^ A strong correlation has been shown between decreased BMD and increased marrow adiposity,^(^ ^51^ ^)^ with increases in marrow adiposity associated with fractures and osteoporosis.^(^ ^52^ ^)^ In an animal BMSC‐transplantation model, BMSCs transplanted from young donors into old recipients resulted in lineage switching from an osteogenic to an adipogenic fate, strongly suggesting that microenvironmental changes with aging play an important role in red marrow conversion.^(^ ^53^ ^)^ As with other causes of osteoporosis, bone loss with aging is a process where the balance of bone remodeling is tipped in favor of bone resorption over bone formation^(^ ^22^, ^54^ ^)^; however, unlike postmenopausal bone loss, age‐related bone loss predominantly reflects decrements in bone formation. Osteoblast differentiation and proliferation is promoted by several transcription factors as discussed previously. In aging, there is a shift from promoting BMSC osteoblastogenesis to favoring adipogenesis caused by decreases in expression of *Runx2* and *Osx*, and an increase in expression of peroxisome proliferator‐activated receptor‐γ.^(^ ^55^ ^)^ WNT signaling is also reported to decrease with age, further reducing osteoblast numbers.^(^ ^56^, ^57^ ^)^

In general, there is a decrease in physical activity with age leading to mechanical unloading and bone loss. Osteocytes are the primary mechanosensing cell type in bone. In a study of long‐term immobilized patients, there were increased levels of sclerostin in their plasma.^(^ ^58^ ^)^ Osteocytes are known secretors of sclerostin, which negatively impact WNT signaling and decrease osteoblast number and activity. Studies in older women have shown an age‐dependent decrease in the number of osteocytes and an increase in empty lacunae,^(^ ^44^, ^59^ ^)^ potentially leading to a decrease in sensing of mechanical stimulation. Important factors limiting the abundance of osteoblasts, osteocytes, and their progenitor cells are the mutually exclusive pathways of cellular senescence^(^ ^60^ ^)^ and apoptosis in these cell types, prominently found in aging bone.^(^ ^61^ ^)^ Stromal cell/osteoblast‐mediated increases in osteoclast differentiation and resorption activity are important potentiators of bone loss. BMSCs from older adults show an age‐dependent increase in expression of *RANKL*, *M‐CSF*, and a decrease in *OPG* expression.^(^ ^62^, ^63^ ^)^ Similarly, osteoblasts from older adults show an age‐dependent increase of pro‐osteoclastic cytokines like IL‐6 and a decrease in *OPG* expression.^(^ ^64^ ^)^ Further promoting the imbalance toward resorption is the decrease in osteoblast number and activity seen with aging.

## Cellular Senescence

The hallmark of cellular senescence is irreversible growth arrest while maintaining cell viability.^(^ ^65^ ^)^ This was first described by Leonard Hayflick, who noted that human embryonic fibroblasts have a finite lifespan in vitro. Further investigations showed that telomeres shorten with each cell division,^(^ ^66^, ^67^ ^)^ forming the basis of the telomere theory of cellular aging with telomere shortening providing the molecular clock. Since those early discoveries, many inducers of cellular senescence have been identified including telomere dysfunction/uncapping, DNA damage, chromatin alterations, reactive oxygen species (ROS), and oncogenes among others.^(^ ^68^ ^)^ Senescent cells are characterized by a stable cell cycle arrest with accompanying morphologic and functional changes, continued metabolic activity,^(69^ ^)^ and resistance to apoptosis through senescent cell antiapoptotic pathways (SCAPs).^(^ ^70^, ^71^ ^)^

Cell cycle arrest in senescent cells is largely the result of two signaling pathways: ATM/p53/ p21^CIP1^ and p16^INK4a^/retinoblastoma. Senescence‐induction stimuli like DNA damage causes kinases such as ataxia telangiectasia mutated (ATM) to phosphorylate and stabilize p53 leading to increases in expression of the cyclin‐dependent kinase inhibitor p21^CIP1^, which promotes cell cycle arrest.^(^ ^72^, ^73^ ^)^ ERK and MAPK signaling upregulates p16^INK4a^ inhibition of CDK4 and CDK6 leading to phosphorylation of retinoblastoma and blocking cell cycle progression from G1 to S.^(^ ^74^, ^75^ ^)^ p16^INK4a^ expression increases in aging cells and tissues and has become an important biomarker for aging studies.^(^ ^76^ ^)^ p21^CIP1^ is thought to be critical for establishing senescence, whereas p16^INK4a^ may maintain the phenotype.^(^ ^73^ ^)^ The roles of p16^INK4a^ and p21^CIP1^ in establishing and maintaining senescence is both cell‐type specific and influenced by the inducing stimuli.^(^ ^77^ ^)^ The cellular processes that lead to senescence include not only cell cycle arrest but inappropriate growth‐promotion pathways^(^ ^78^ ^)^ such as mTOR.^(^ ^79^, ^80^ ^)^ It is important to note that senescence is not restricted to only mitotic (proliferating) cells but also to mostly nondividing postmitotic cells, such as osteocytes.^(^ ^60^ ^)^

Adult cells in various tissues respond differently to damage and can be prone to either senescence or apoptosis. These choices can lead to an appropriate mechanism for renewal of the tissue such as epithelial cell apoptosis^(^ ^81^ ^)^ or senescence of stromal cells.^(^ ^82^ ^)^ However, most tissue repair occurs through stem cell proliferation and differentiation. These stem cells also undergo senescence with physiologic aging, limiting their proliferation and differentiation capability,^(^ ^83^ ^)^ as well as responsiveness to external signals.^(^ ^84^ ^)^ The decline in function of differentiated cells and their stem cell pools are an important driver of age‐related pathologies.

### Inducers and biomarkers of cellular senescence in bone

The discovery of pathways that induce senescence has led to the identification of biomarkers for the detection of senescent cells that will continue to expand and increase in specificity as tools for further investigation into aging processes.

#### *Telomere dysfunction*

Telomeres are repeated DNA sequences found at the end of chromosomes, which are protected by a cap of proteins and maintained by telomerase. The process of replicative cell division causes the loss of telomere length, which increases with age.^(^ ^85^ ^)^ This leads to the activation of the DNA damage repair (DDR) system that recognizes the end of the telomere as a double‐strand break,^(^ ^86^ ^)^ and further activates p53/p21^CIP1^ and p16^INK4a^ to cause withdrawal of the cell from the cell cycle and promote senescence.^(^ ^87^ ^)^ These observations led to the idea that telomere dysfunction‐induced foci (TIFs), representing the colocalization of DDR proteins to the end of telomere, can determine cell proliferation and differentiation capabilities. It has become a useful marker for senescent cells and has been used in studies of various bone cell types including BMSCs and osteocytes.^(^ ^60^, ^88^ ^)^ Interestingly, postmitotic osteocytes, like muscle cells and neurons, may form shortened telomeres in a replication‐independent manner.^(^ ^89^ ^)^

Werner syndrome (WS) and dyskeratosis congenita are two genetic diseases with features of premature aging exhibiting shortened telomeres and premature osteoporosis. A model of accelerated aging in mice targeting the WS helicase and telomerase recapitulates the low bone mass and age‐related osteoporosis seen in affected individuals and further shows shortened osteoblast lifespan and impaired differentiation without impaired osteoclast differentiation.^(^ ^88^, ^90^ ^)^ This would indicate that replicative aging of osteoblast precursors plays a pivotal role in senile osteoporosis. Further knockout studies in mice that eliminate only telomerase reverse transcriptase showed a decrease in bone mass with reduced osteoblast differentiation but increased osteoclastogenesis.^(^ ^91^ ^)^ The signaling pathway mediating this effect was shown to involve increased p53 expression with cell cycle arrest, apoptosis, and a decrease in Runx2 expression.^(^ ^88^ ^)^ As further confirmation of these studies, overexpression of telomerase in BMSCs maintains their osteogenic differentiation capabilities in vitro while allowing increased bone formation in vivo.^(^ ^92^, ^93^, ^94^, ^95^ ^)^ In a study where circulating leukocytes were isolated from over 2000 women, it was found that telomere length was significantly correlated with BMD, and shorter telomeres were found in women with clinical osteoporosis.^(^ ^96^ ^)^ This was directly contradicted by another study reporting that leukocyte telomere length was not associated with BMD in aged men and women.^(^ ^97^ ^)^ Further studies are required to understand how telomere length or dysfunctional telomeres could be used as potential diagnostic markers to predict age‐related osteoporosis.

#### *DNA damage*

DNA damage occurs by exposure to external factors such as ultraviolet and ionizing radiation, as well as endogenous factors like ROS and metabolic byproducts. The cellular DDR uses DNA‐damage checkpoints to control cell cycle progression. As part of DDR signaling, p53 is stabilized, promoting cell cycle arrest through p21^CIP1 (^ ^72^ ^)^ and secondarily through p16^INK4a^.^(^ ^60^, ^98^ ^)^ In the case where damage cannot be repaired, p53 induces cellular senescence.^(^ ^99^ ^)^ Also upregulated by DDR is the zinc finger transcription factor GATA4, which stimulates NF‐κB and senescence‐associated secretory phenotype (SASP) production, further reinforcing the senescent phenotype.^(^ ^100^ ^)^ A mouse study deleting an endonuclease (excision repair cross‐complementary group 1‐xeroderma pigmentosum group F) important for DDR, which is well conserved in a human progeroid syndrome deficiency causing osteopenia, osteoporosis, and abnormal skeletal development, showed decreases in osteoprogenitor cells with an increase in DNA damage as evidenced by increases in γ histone family member X (γH2AX foci, phosphorylation of the Ser‐139 residue of the histone variant H2AX), senescence, and SASP of BMSCs and osteoblasts.^(^ ^101^ ^)^ In another study, it was found that old mice had significantly increased markers of DNA damage such as γH2AX foci and senescence including G1 cell cycle arrest, phosphorylation of p53, and increased levels of p21^Cip1^, as well as an increase in GATA4 and activation of NF‐κB stimulated SASP secretion.^(^ ^102^ ^)^ These studies implicate a role for DNA damage‐induced senescence limiting the number of osteoprogenitors and osteoblasts with aging and an increase in SASP facilitation of osteoclastogenesis.

#### *Chromatin alterations*

The study of epigenetic changes with aging and its influence on gene transcription, proliferation, and DNA damage is a complex process entering an exciting era of discovery. The loss of heterochromatin with aging has been documented from *Caenorhabditis elegans* to humans including important, normally heterochromatic areas such as telomere ends and pericentromeric regions.^(^ ^103^ ^)^ Interestingly, in senescent cells there are also new regions of heterochromatin termed senescence‐associated heterochromatic foci (SAHF).^(^ ^104^, ^105^ ^)^ These regions are transcriptionally inactive and may function to assist in the cell cycle arrest that occurs in senescence.^(^ ^104^, ^106^, ^107^ ^)^ SAHF may not be a consistent marker as it is not found in all senescent cells such as those isolated from patients with Hutchinson–Gilford progeria syndrome.^(^ ^108^, ^109^ ^)^ Another marker found in all senescent cells to date is senescence‐associated distension of satellites (SADS), which detect unraveled pericentromeric satellite heterochromatin. It is not exclusive to either p16^Ink4a^ or p21^Cip1^ pathways^(^ ^110^, ^111^ ^)^ in contrast to SAHF, which is linked to p16‐pathway activation.^(^ ^104^ ^)^

Changes in heterochromatin detected with aging are caused in large part to nucleosome remodeling, which is determined by the histone compliment and its modifications termed marks. There is an overall loss of histones with aging and a change in the abundance of histone variants present, their location, as well as modifications such as methylation, acetylation, and phosphorylation.^(^ ^103^ ^)^ With aging in general, there is an increase in activating histone marks (like H3K4me3) and a decrease in repressive marks (like H3K9me3),^(^ ^112^ ^)^ but the picture is much more complex because there are differences even noted between cells from the same donor, as well as those from monozygotic twins.^(^ ^113^ ^)^

Several studies in BMSCs have investigated the role of heterochromatin dysfunction and its promotion of senescence. WS is a progeria caused by mutation in the WRN gene, which encodes a highly conserved helicase important for many functions related to DNA repair and telomere maintenance.^(^ ^114^, ^115^ ^)^ WRN^–/–^ BMSCs when passaged show premature loss of replication, telomere length, senescence with DNA damage, and a significant decrease in the repressive H3K9me3 mark important for maintenance of heterochromatin.^(^ ^116^ ^)^ Coimmunoprecipitation assays show an association between WRN SUV39H1 (a methyltransferase for H3K9me3), HP1α, and LAP2β, with the latter two proteins helping to associate the heterochromatin with the nuclear envelope. Knockdown of SUV39H1 or HP1α in BMSCs led to decreased H3K9me3 expression and senescence linking heterochromatin destabilization with senescence.^(^ ^116^ ^)^ In a study done in mice, A‐type lamins were shown to interact with methyltransferase Suv39h1, depletion of which reduces H3K9me3 levels, restores DNA repair capacity, and delays senescence in progeroid cells. Furthermore, in Zmpste24^‐/‐^ mice, a lamin A‐deficient model of progeria, loss of Suv39h1 delays weight loss with increased BMD and was shown to extend lifespan by 60%.^(^ ^117^ ^)^ Corroborating the mice data, human BMSCs derived from the dental pulp of aged patients had reduced levels of SUV39H1, H3K9me3, and the RecQ DNA helicase WRN.^(^ ^116^ ^)^ Other investigations have shown that polycomb group (PcG) proteins are important for binding to target genes, recruiting EZH2 methyltransferase, methylating target histones like H3K27me3 and binding to condensed nucleosomes.^(^ ^118^ ^)^ In addition, EZH2 methyltransferase is associated with the regulation of osteogenesis and bone development.^(^ ^119^, ^120^, ^121^, ^122^ ^)^ As a counterbalance, JMJD3 demethylase, whose expression is increased in response to oncogenes or stress, demethylates H3K27me3 decreasing PcG complex binding leading to increased expression of p16^INK4A^ and senescence.^(^ ^123^, ^124^ ^)^ These studies highlight the impact changes in heterochromatin regions can have on senescence promotion that in turn lead to a decrease in bone marrow progenitor cells capable of renewal with aging.

Globally DNA methylation declines with aging. For example, Alu hypomethylation was associated with advancing age and reduced BMD.^(^ ^125^ ^)^ Additionally, in BMSCs there is a decrease in the expression of DNA methyltransferases DMNT‐1 and DNMT‐3B with aging and their inhibition induces senescence.^(^ ^126^, ^127^ ^)^ The loss of DMNTs increases expression of p16^INK4A^ and p21^CIP1^ but decreases expression of PcG proteins.^(^ ^126^ ^)^ In contrast, there is an increase in methylation of promoter‐associated CpG islands near genes important for differentiation, cell‐type–specific functions, and transcription factors and their modulators.^(^ ^112^, ^128^ ^)^ Studies of methylation at CpG sites in BMSCs showed similar changes in methylation from in vitro and in vivo samples, indicating that hypermethylated sites corresponded to homeobox family genes important for differentiation and expression of Runx2 and DLX5, important transcription factors for osteogenic differentiation.^(^ ^129^ ^)^ Progress in mapping DNA methylation patterns in specific tissues is building the “epigenetic clock,” with the potential to create a powerful database for aging studies.

In a study of older women with underlying osteoporosis and osteoarthritis, where the bone samples from the femoral head were analyzed, the overall methylation levels at CpG loci were well correlated between the two age‐associated comorbidities.^(^ ^130^ ^)^ However, careful characterization and genomewide methylation profiling of bone samples identified unique methylation sites linked to cell differentiation and osteogenesis in the osteoporotic and osteoarthritic populations.^(^ ^131^ ^)^ In a study where DNA methylation was used as a predictor for osteoporosis in blood samples from different aged patients, no such correlation was achieved, suggesting that peripheral blood is not a good source from which to make predictions about changes in bone with aging.^(^ ^132^ ^)^ In another study in postmenopausal women, osteoporosis chromatin modifying enzymes HAT1, KAT5, HDAC6, MBD1, and DNMT3A were all downregulated, with a direct correlation between abundance of HAT1, HDAC6, and MBD1 and bone quality.^(^ ^133^ ^)^ A comparative study with age‐related osteoporosis is still elusive, leaving the question open as to whether the changes in these epigenetic enzymes during postmenopausal osteoporosis were mainly caused by the loss of estrogen.

#### *Reactive oxygen species*

ROS are beneficial at low levels as they promote osteoclast differentiation and resorption of bone and are usually in a delicate balance with antioxidants.^(^ ^134^ ^)^ However, when levels of ROS increase above beneficial levels, as with aging, they cause cell death in osteoblasts and osteocytes and the destruction of bone by promoting osteoclast differentiation and activity.^(^ ^135^ ^)^ Human studies show increases in ROS and decreases in antioxidant levels correlate with increases in osteoclast activity and decreases in osteoblast activity as a function of age and osteoporosis.^(^ ^134^, ^136^, ^137^ ^)^ Osteoclasts have the largest number of mitochondria of any cell type because of the energy and acid production necessary for resorption. These mitochondria and NADPH oxidases are important sources for ROS. In a mouse study of osteoclasts expressing a mitochondria‐targeted catalase, there were increases in bone mass and decreases in osteoclast formation and survival that even protected women from ovariectomy‐induced bone loss, highlighting the function of mitochondrial‐produced ROS.^(^ ^138^ ^)^ Mitochondrial defects accumulate with age both morphologically and functionally, including decreases in biogenesis, mitochondrial dysfunction, and bioenergetic failure. All of these defects help to drive senescence by the interplay of mitochondrial dysfunction/ROS production with the DDR and aberrant signaling through telomere shortening/replicative senescence pathways, with the ultimate effect of disrupting bone cell and especially stem cell function.^(^ ^139^ ^)^

#### *microRNAs*

miRNAs, as previously introduced, play an important role in bone remodeling; changes in their abundance with age can greatly affect and potentiate senescence. Their importance in regulating senescence was shown when ablation of the RNase III family endoribonuclease Dicer, essential for the maturation of most miRNAs, caused senescence.^(^ ^140^ ^)^ miRNA‐195, which directly targets the 3′ untranslated region of telomerase reverse transcriptase (TERT), increases with age.^(^ ^141^ ^)^ Furthermore, overexpression of miR‐195 induced BMSC senescence, whereas a knockdown of miR‐195 increased TERT expression and promoted telomere relengthening.^(^ ^141^ ^)^ Another example of miRNA modulation of the senescence machinery comes from studies of DNA methyltransferase‐1 on repression of the RANKL promoter. It was found that TNFα could lift this repression by upregulation of miR‐140‐3p and miR‐126.^(^ ^142^ ^)^ Two recent studies have shown miRNAs are at the critical switching point between BMSC osteogenesis and adipogenesis/senescence. In one, increases in miR‐188 downregulated histone deacetylase 9 and RPTOR‐independent companion of MTOR complex causing bone loss and fat accumulation^(^ ^143^ ^)^; in the other, miR‐363‐3p targeted TNF receptor‐associated factor 3 with similar effect.^(^ ^144^ ^)^ Other roles for miRNA and senescence induction include regulation of IGF‐1 signaling, ROS, and stem cell exhaustion.^(^ ^145^ ^)^ miRNA levels are now being correlated as biomarkers for diseases such as osteoporosis.^(^ ^146^, ^147^ ^)^ One report found a direct correlation between miR‐29b‐3p and improved bone formation rate/bone surface,^(^ ^148^ ^)^ with declines in miR‐29b‐3p, among others, predicting changes in bone fragility, fracture rates, and bone turnover in postmenopausal women.^(^ ^149^ ^)^ Retinoic acid receptor‐related orphan receptor β (Rorbeta, Rorb) is a gene that is highly upregulated in aged mice and human bone samples, but is significantly downregulated during the progression of osteogenesis, and is itself downregulated by direct binding by miR‐219a‐5p, thus making miR‐219a‐5p another potential marker for future predictions of age‐related osteoporosis.^(^ ^150^ ^)^

Additional biomarkers of senescence include senescence‐associated β‐galactosidase (SA‐βgal)^(^ ^151^ ^)^ and senescence‐associated α‐fucosidase,^(^ ^152^ ^)^ lysosomal enzymes that correlate with, but do not have a causative effect on senescence. In addition, cellular senescence induces changes in nuclear morphology with a progression from several compact nucleoli of proliferating cells to one enlarged nucleolus in senescent cells stuck at the G1/S stage of cell cycle progression.^(^ ^108^, ^153^, ^154^ ^)^ There is also diminished ribosome biogenesis and accumulation of rRNA precursors with senescence.^(^ ^108^ ^)^ Ribosomal protein L29 may be a related biomarker of senescent cells.^(^ ^108^ ^)^ Single markers of senescence are limited and may not be accurate under certain conditions,^(^ ^155^, ^156^, ^157^ ^)^ but a combination of several markers has been shown to be reliable for detecting cellular senescence.^(^ ^74^ ^)^

### Senescence‐associated secretory phenotype

Senescent cells are metabolically active and secrete signaling factors (cytokines, chemokines, and growth factors), proteases (matrix metalloproteases and serine proteases), extracellular matrix proteins, nonprotein components (reactive oxygen and nitrogen species), and extracellular vesicles containing miRNA.^(^ ^158^ ^)^ These collectively are referred to as the SASP.^(^ ^159^ ^)^ Recent studies indicate that components of the SASP may vary depending on whether they are derived from cells early or late after the onset of senescence.^(^ ^160^, ^161^, ^162^ ^)^

Multiple inducers of senescence, the timing of senescence induction, and differing cell types lead to unique profiles for the SASP that can affect neighboring cells as well as those at a distance. The SASP has both beneficial and negative impacts on biological processes. The beneficial aspects include wound healing,^(^ ^163^ ^)^ embryogenesis,^(^ ^164^, ^165^, ^166^ ^)^ and tumor suppression by reinforcing growth arrest.^(^ ^167^, ^168^ ^)^ After damage in a young organism, senescent cells secrete SASP factors, which can alert the immune system for their clearance and promote tissue regeneration through increased stemness in surrounding cells followed by proliferation and differentiation.^(^ ^169^, ^170^ ^)^ The temporary presence of senescent cells in tissues and their benefits shift as aging progresses. Senescent cells accumulate with aging, leading to chronic production of SASP components and inflammation.^(^ ^171^ ^)^ Chronically, the SASP can inhibit proliferation and promote senescence in nearby cells.^(^ ^167^, ^172^, ^173^ ^)^ This can lead to a prolonged dedifferentiated state that blocks tissue regeneration after damage.^(^ ^174^ ^)^ A persistent SASP can also be protumorigenic in committed precancerous cells promoting their proliferation, survival, and metastasis.^(^ ^175^ ^)^ The increase in senescent cells and their SASP with aging leads to dysfunctional tissue and disease states.

### Targeting cellular senescence in bone

Recently, studies were undertaken to identify senescent cells and their SASP in the bone microenvironment.^(^ ^60^ ^)^ Cells were isolated from the bone of young (6‐month‐old) and old (24‐month‐old) mice and magnetic‐activated cell sorting was used to enrich in vivo cell types. The data indicated that *p16* ^*Ink4a*^ mRNA expression increased with aging in myeloid cells, B and T cells, osteoblast progenitors, osteoblasts, and osteocytes.^(^ ^60^ ^)^ There was an accumulation of senescent osteocytes in older mice identified by increased SADS and TIF staining. In this study, SASP mRNA profiles of 36 previously identified SASP factors were investigated. Few changes in SASP factors were seen with aging in osteoblast progenitors, osteoblasts, and B and T cells, but in myeloid and osteocyte cells greater than 23 out of 36 SASP factors significantly increased.^(^ ^60^ ^)^ Consistent with the data obtained from the mouse model, small needle bone biopsies from young and older postmenopausal women showed increases with aging in *p16* ^*INK4A*^ and *p21* ^*CIP1*^, as well as 12 of the 36 SASP factors investigated.^(^ ^60^ ^)^ The increase with aging in senescent mouse osteocytes and SASP has been confirmed by other investigators and extended to show increased levels of RANKL in associated age‐dependent cortical bone loss.^(^ ^176^ ^)^ In studies of mouse osteoprogenitors, it was found that their numbers decline more than 50% with aging and are positive for markers of DNA damage, increased *p21* ^*Cip1*^, and elevated expression of SASP genes.^(^ ^102^ ^)^ Interestingly, elimination of osteoclasts in mice has no effect on bone loss with aging.^(^ ^177^ ^)^

**Fig 1:**
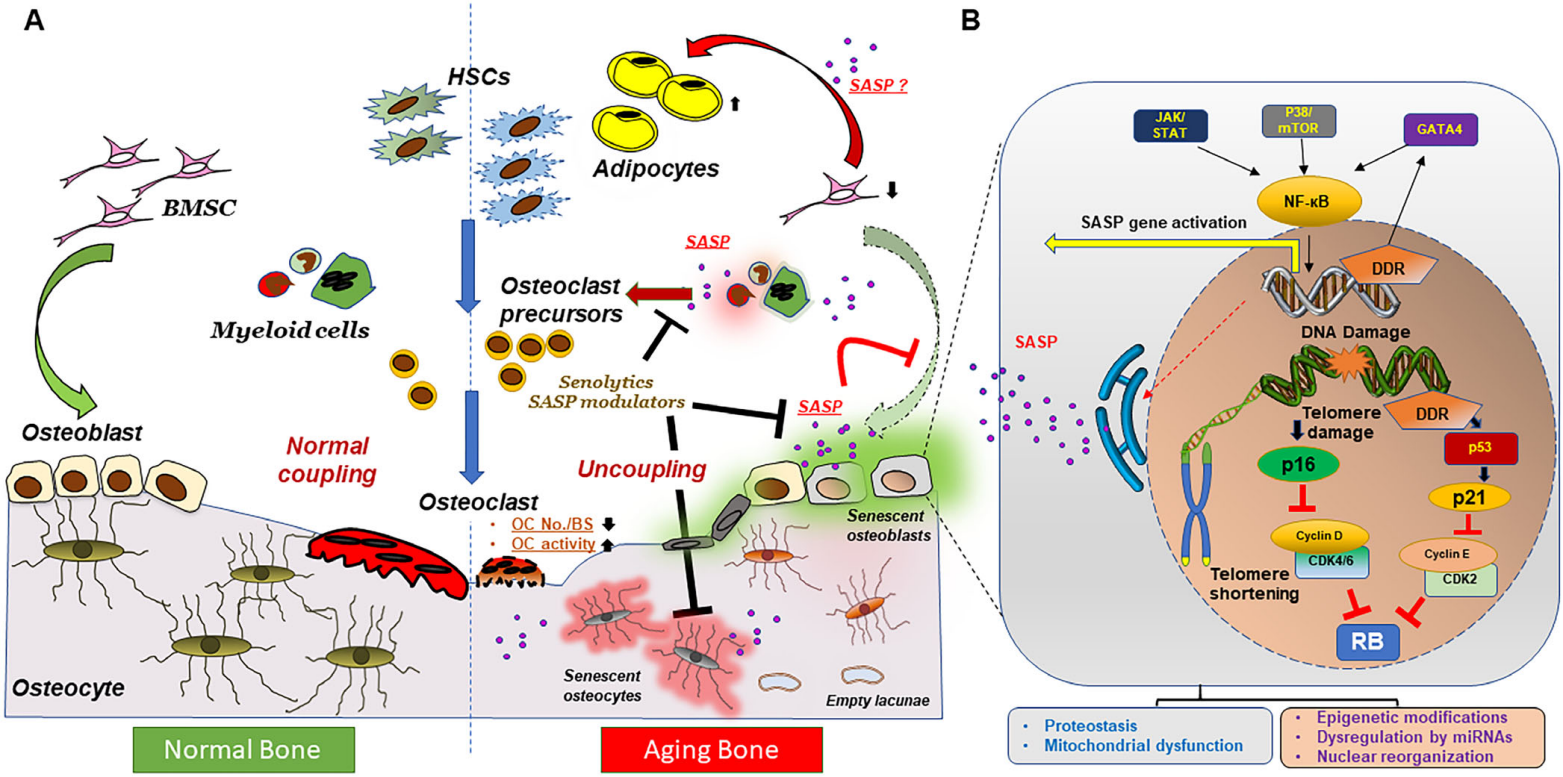
Overview of aging bone and the cellular processes regulating senescence and the SASP. (*A*) Normal bone formation involves activation of BMSCs to differentiate into osteoblasts, which fill in resorption pits created by osteoclasts. The release of matrix‐embedded factors by resorption in turn activates osteoblast proliferation, maturation, and differentiation, creating a coordinated homeostatic process known as “coupling.” Aging induces dysfunctional homoeostasis leading primarily to reduced bone formation and mineralization but also increased resorption by osteoclasts owing to increased osteoclast precursors. However, the overall osteoclast numbers on the bone surface decline with aging. BMSCs have a reduced capacity to form osteoblasts, and preferentially differentiate into adipocytes. Osteocytic cell death and empty lacunae promote the loss of the canalicular network. Senescent bone cells and their proinflammatory secretome (i.e., SASP) have been shown to play key roles in propagating the alterations seen in the aging bone. Targeted killing of senescent cells can be achieved by drugs called *senolytics* and inhibition of the SASP by senomodulators (or senomorphics). (*B*) Taking senescent osteoblasts as an example of a generalized senescent cell, several subcellular processes may become drivers of cellular senescence. Shortened or damaged telomeres or other DNA damage followed by the DDR activates cell cycle inhibitory pathways regulated by p16^INK4a^ or p21^CIP1^, which then suppresses cyclins such as cyclin D, CDK4/6, cyclin E, and CDK2. Together with the activation of the DDR, several pathways regulated by GATA4, mTOR, or JAK/STAT proteins activate the NF‐κB–based transcriptional activation of proinflammatory SASP proteins, which in turn are excreted by the cell to induce deleterious autocrine, paracrine, or endocrine responses. Mitochondrial dysfunction, proteostasis, epigenetic modifications to the chromatin, miRNA‐based dysregulation of genes and changes in the nuclear lamina are other known characteristics of a senescent cell. BMSCs, bone mesenchymal stem cells; BS, bone surface; DDR, DNA damage repair; HSCs, hematopoietic stem cells; miRNAs, microRNAs; OC, osteoclast; RB, retinoblastoma gene product; SASP, senescence‐associated secretory phenotype.

To evaluate the role of senescent cell accumulation and SASP in bone aging, several approaches have been taken to eliminate senescent cell burden in mice. These include using a genetic model with the INK‐ATTAC transgene containing a FKBP‐caspase‐8 fusion protein (which is lethal to p16^+^ cells with administration of the drug AP20187) or a drug combination of dasatinib and quercetin (D + Q) that clears senescent cells.^(^ ^178^ ^)^ Mice were treated starting at 20 months of age when bone loss was documented. Both approaches showed partial elimination of senescent osteocytes with concomitant higher bone mass, strength, and microarchitecture in comparison with controls. Furthermore, these approaches suppressed bone resorption and maintained osteoblast numbers and bone formation in trabecular and cortical bone.^(^ ^178^ ^)^ The JAK inhibitor (JAKi) ruxolitinib suppresses components of the SASP, particularly IL‐6, Il‐8, and PAI‐1,^(^ ^179^, ^180^ ^)^ which are known to be important for osteoclastogenesis and bone resorption.^(^ ^178^ ^)^ JAKi treatment of 22‐month‐old mice for 2 months yielded similar results to those of the INK‐ATTAC transgene strategy and senolytic treatments, with lower osteoclast numbers and no significant differences in osteoblast numbers.^(^ ^178^ ^)^ In vitro studies further confirmed that senescent cell‐conditioned media promotes osteoclastogenesis.^(^ ^178^ ^)^ Although senescent osteoprogenitors, osteoblasts, and osteocytes have direct consequences on bone architecture, the role of other senescent populations (such as myeloid‐lineage cells) in aging bone are still to be determined. The identification of senescent osteocytes and osteoblasts in vivo and the rescue of age‐related bone loss by the elimination of senescent cells indicate a pivotal role for cellular senescence and their SASP in bone loss and osteoporosis with aging (Fig. 1).

Another model of genetic clearance of p16 cells (i.e., p16‐3MR mice), failed to show any recovery of the aging bone phenotype and the elimination of senescent osteoclast progenitors did not affect the endocortical osteoclast number and age‐associated bone loss.^(^ ^177^ ^)^ However, unlike studies in INK‐ATTAC mice,^(^ ^178^ ^)^ those performed in the p16‐3MR model failed to clear senescent osteocytes but did clear senescent osteoclast progenitors.^(^ ^177^ ^)^ Moreover, elimination of senescent osteoclast progenitors did not have any significant change on the bone architecture of the aged p16‐3MR mice, suggesting senescent osteocytes are key drivers for age‐related osteoporosis. Taken together, these studies indicate that cellular senescence is a key mechanism that contributes to age‐related osteoporosis. In addition, oxidative stress and DNA damage linked to ROS are now being associated with various other pathways of age‐related osteoporosis.^(^ ^181^ ^)^ For example, vitamin D insufficiency is associated with senescence‐associated age‐related osteoporosis,^(^ ^182^ ^)^ and recent studies suggest that it protects against age‐related osteoporosis via a VDR‐EZH2‐p16 axis.^(^ ^183^ ^)^

### Radiation‐induced bone loss: cellular senescence and similarities to age‐related osteoporosis

Ionizing radiation (IR) is present all around us and though levels of IR in the atmosphere are submaximal, ultraviolet light from the sun imparts significant radiation that produces DNA damage by direct and/or indirect methods such as the generation of free radicals and ROS. Therapeutic IR used for cancer treatment targets both normal and cancer cells to induce apoptosis, autophagy, or senescence. It is still unclear if there is a threshold of IR that induces a different cell fate. Although the cell has mechanisms to counter small levels of IR using antioxidants, catalase, superoxide dismutase, glutathione peroxidase, and reduced glutathione, an excess dose of IR may overcome these defense mechanisms to induce DNA damage.^(^ ^184^ ^)^ IR‐induced single‐strand breaks and double‐strand breaks (DSBs) in the DNA produce a DDR. The DDR induces a growth arrest and failure to repair damaged DNA leads to a permanent state of growth arrest consistent with cellular senescence with the expression of *p21* ^*CIP1*^ and *p16* ^*INK4A*^, as well as the SASP. It has been noted that cells, which expressed the senescence marker *p16* ^*Ink4a*^ in the absence of a DDR, did not have a SASP.^(^ ^185^ ^)^ Thus, the cellular state, including DDR, and not just the expression of markers of senescence, drives the senescent phenotype.

There are six major DNA repair pathways, O6‐methyl guanine methyltransferase, base excision repair, nucleotide excision repair, mismatch repair, and DNA DSB repair, which includes homologous recombination (HR) and nonhomologous end‐joining (NHEJ). Although IR‐induced cellular senescence is known to be triggered by the NHEJ pathway initiated by a DSB, the indirect effects of IR, which may also lead to DNA damage, is repaired by HR. The DSB component, mediated by ATM, leads to phosphorylation of the DDR mediator H2AX. Phosphorylated γH2AX then initiates the recruitment of other proteins of the DDR. The number of γH2AX foci equates to DSB sites in a nucleus and although IR‐induced γH2AX foci formation seems to resolve over time, the ability of cells to resolve these lesions depends on the DDR components, Ku70 and DNA‐PKC, which are both key players in the NHEJ mechanism. Both Ku70 and DNA‐PKC are high turnover proteins and their levels following IR determine the fate of DSB repair. Stabilization of these proteins either by activation of the cAMP–WNT pathway or by suppression of proteasome‐based degradation allows radiated osteoprogenitors, osteoblasts, and osteocytes to survive the IR‐induced DNA damage and cellular apoptosis and ultimately leads to bone accrual in rodent studies.^(^ ^186^, ^187^, ^188^ ^)^ However, the major outcome of IR‐induced damage on bone cells that sustain unresolved DNA damage is cellular senescence.

Although cellular senescence was originally proposed as an explanation for the limited replicative potential of a cell, IR leads to stress‐induced premature senescence (SIPS). Different forms of IR such as X‐rays or γ‐rays lead to SIPS.^(^ ^184^ ^)^ Induction of SIPS by IR may vary by dose: A low dose may cause DNA damage through oxidative damage without generating a senescence phenotype, whereas high‐dose IR causes DNA damage through DSBs leading to sustained DNA damaged sites, often in the telomere, and then to senescence.

Low‐dose IR‐induced DNA damage in cells is mainly repaired and osteoprogenitors such as BMSCs do respond to low‐dose IR by undergoing growth arrest,^(^ ^189^ ^)^ with DNA damage lesions not passed to the daughter BMSCs.^(^ ^190^ ^)^ Apart from these reports, most studies suggest that BMSCs are largely resistant to IR, based in part on the abundance of nuclear lamina proteins. Lamin A is a major component of the nuclear lamina, and the ability of cells to resist DNA damage from IR has been attributed to lamin A content. BMSCs are known to have one of the highest lamin A levels of any cell type, providing stiffness to the cells^(^ ^191^ ^)^ and potentially resistance to SIPS. In contrast, several lines of in vitro evidence have suggested that BMSCs do not lose their ability to proliferate and form colonies in response to low‐ and high‐dose IR^(^ ^188^, ^189^ ^)^ but have a reduced osteogenic potential.^(^ ^192^, ^193^ ^)^ The in vivo evidence that BMSCs undergo IR‐induced senescence is limited. In highly senescent environments, such as high‐dose IR and aging, BMSCs tend to differentiate into adipocytes^(^ ^188^ ^)^ a phenomenon commonly observed in skeletal regions where patients receive radiotherapy for cancer treatment.^(^ ^194^ ^)^

Previous studies show that sustained DNA damage to osteoblasts and osteocytes several weeks post‐IR greatly impaired bone formation.^(^ ^187^, ^188^ ^)^ However, there were stark differences between the cells that showed DNA damage and the cells that entered the programmed cell death pathway, with the percentage of apoptotic cells accounting for only a small fraction of cells that showed DNA lesions.^(^ ^188^ ^)^ This gap in understanding was recently bridged by studies showing that bone cells undergo senescence in response to IR. Cellular senescence was assessed by gene expression analysis of the senescence markers *p21* ^*waf1/Cip1*^ and *p16* ^*Ink4a*^, as well as SA‐βgal staining and DSB assessment at telomere sites (TIFs).^(^ ^195^ ^)^ Interestingly, *p21* peak expression was predominantly higher than *p16* ^*Ink4a*^ expression on day 1 post‐IR, whereas *p16* ^*Ink4a*^ expression peaked around day 42 post‐IR. The gene expression data were confirmed by histological analysis of senescent cells using SA‐βgal staining on day 28 and by TIF assay on day 42 post‐IR.^(^ ^195^ ^)^ Senescent bone‐lining cells, osteocytes, and bone marrow cells together indicated an overall senescent environment, accompanied with a SASP profile. Pharmacologic clearance of senescent cells using senolytic drugs relieved the senescent cell burden and SASP, and ultimately alleviated IR‐related bone loss as well. Further studies are required to understand the role of different cell types and cellular pathways that contribute to the IR‐induced suppression of bone formation. For example, it is still unclear if the vasculature system in bone undergoes the same degree of IR‐induced cellular senescence as other bone cells, and if that directly or indirectly contributes to bone loss.

### Sex steroids and skeletal health

Several studies have attempted to understand the role of gonadal sex steroids in regulating skeletal health. Current understanding indicates that estrogen may play a role in the bone health of both postmenopausal women^(^ ^196^ ^)^ and aging men.^(^ ^197^, ^198^ ^)^ Although the role of testosterone in regulating BMD in men was not consistent between studies,^(^ ^197^, ^198^ ^)^ some studies found a direct correlation with declining testosterone levels with loss in BMD,^(^ ^199^, ^200^ ^)^ and treatment with testosterone has shown improvement in trabecular BMD in the spine.^(^ ^201^ ^)^ However, androgens may indirectly regulate skeletal maintenance by aromatization into estrogen and binding to the estrogen receptor.^(^ ^202^ ^)^ In evaluating the role of estrogen deficiency in aging and osteoporosis, independent roles for estrogen function in age‐associated bone loss were identified that did not overlap with cellular senescence‐related bone loss.^(^ ^203^ ^)^ In these studies, short‐term estrogen treatment in human subjects failed to affect markers of senescence or the SASP.^(^ ^203^ ^)^ Preclinical genetic studies with clearance of p16–positive senescent cells in mice alleviated age‐related osteoporosis,^(^ ^178^ ^)^ but failed to alleviate ovariectomy‐induced bone loss.^(^ ^203^ ^)^ This clearly indicated that estrogen deficiency‐related bone loss is independent of senescence‐related bone loss. However, further studies are needed to better ascertain the relationships between gonadal sex hormone deficiencies and cellular senescence pathways, especially in clinical studies.

### Pharmacologic targeting of senescent cells in the treatment of osteoporosis

Preclinical studies indicate a possible role for senotherapeutic agents in the treatment of age‐related osteoporosis.^(^ ^178^ ^)^ Such agents target senescent cell apoptotic pathways and result in senescent cell clearance (i.e., senolytics) or reduction of the SASP, leaving senescent cells intact (i.e., senomodulators). Because of the accumulation of senescent cells in other types of osteoporosis, such as radiotherapy‐induced bone loss, senotherapeutic approaches may also be effective in these clinical scenarios. Because there is also some evidence for senescent cell burden in mechanisms of bone loss related to diabetes mellitus, skeletal unloading, glucocorticoid use, and perhaps androgen insufficiency,^(^ ^204^ ^)^ the therapeutic umbrella for senolytic and senomorphic drugs in osteoporosis may be further expanded.

Although both senolytics and senomodulators may have similar beneficial effects in preclinical animal models of bone loss, pharmacodynamics and toxicity considerations in humans may dictate their future use as therapy for osteoporosis. Senolytic agents can likely be given once or consecutive times over a few days every 4–6 weeks based on estimations of senescent cell turnover and thus the time to reaccumulation of senescent cells.^(^ ^205^, ^206^ ^)^ Senomodulators would need to be given more frequently, probably daily, increasing the likelihood of adverse drug events compared with senolytics. Possible drug resistance would not be an important consideration because senescent cells are nondividing. Also, immunological senescent cell clearance, which normally occurs in youth and thereafter diminishes,^(^ ^207^, ^208^, ^209^, ^210^ ^)^ may be improved with senotherapeutics and contribute to their overall beneficial effects. Compared with current pharmacologic treatment considerations for osteoporosis, senolytics offer the possibility of long‐term treatment.

It is still unclear to what extent, if any, postmenopausal osteoporosis may be mediated or amplified by senescent cells in the bone microenvironment or at distant locations. Preclinical studies in ovariectomized mice suggest that acute estrogen deprivation does not promote accumulation of senescent cells in bone.^(^ ^203^ ^)^ In support of this observation, short‐term administration of estrogen in postmenopausal women does not reduce markers of senescence in bone biopsies.^(^ ^203^ ^)^ Despite these findings, treatment of postmenopausal women with senolytics may still improve bone parameters, such as bone turnover markers and BMD, and this is currently being evaluated for the senolytic cocktail D + Q and for Fisetin (ClinicalTrials.gov Identifier: NCT04313634, Targeting Cellular Senescence With Senolytics to Improve Skeletal Health in Older Humans). Ovariectomized mice are limited as models of postmenopausal osteoporosis because the typical paradigm for study involves estrogen depletion in young animals and only for short periods. In the setting of possible cellular senescence‐based contributions to postmenopausal bone loss (and consideration for the use of senotherapeutic agents), both estrogen deficiency and aging must be considered as cumulative or synergistic effects.

Another area for future therapeutic interventions in age‐related osteoporosis is combination therapy with senotherapeutic agents and existing approved drugs for osteoporosis. Although senotherapeutic interventions in preclinical studies suggest that their primary effect is on bone formation (with secondary effects on inhibition of bone resorption), complementary and/or additive effects of current anabolic agents or antiresorptive drugs may hold future promise. In fact, at least one medication used for the treatment of bone loss (zoledronate) conveys a mortality benefit independent of preventing fracture, as well as an extension of lifespan in an animal model of accelerated aging.^(^ ^211^, ^212^, ^213^, ^214^ ^)^ Also, zoledronate was able to extend the lifespan of human mesenchymal stem cells by protecting against the accumulation of DNA damage, an important mechanism of cell aging, and preserving their ability to proliferate and differentiate in vitro.^(^ ^215^ ^)^ Thus, zoledronate itself may have senotherapeutic properties.

## Conclusions

Substantial alterations in bone architecture occur with aging, including decreases in trabecular thickness and number, cortical bone loss and porosity, and increase in marrow adiposity. These changes reflect imbalances in bone remodeling, favoring a net loss of bone caused predominately by increased osteoclast activity in postmenopausal women, as well as both poor bone formation and increased osteoclast activity in older men and women. Cellular senescence and apoptosis of osteoblasts and osteocytes account for much of the aging phenotype in bone, although appear to be independent of estrogen‐mediated effects.

There is growing evidence to suggest that cellular senescence in bone can be triggered by ROS, DNA damage, telomere dysfunction, and heterochromatin changes, depending on the cell type. miRNAs serve to modulate critical switching points, such as those between osteogenesis and adipogenesis, and aspects of the senescence program. The SASP likely mediates local and even distant deleterious effects of senescent cells, especially by myeloid cells and osteocytes. Radiation‐induced bone loss provides an accelerated aging bone model that recapitulates many aspects of age‐related bone loss. With both physiological and premature bone aging, genetic and pharmacologic approaches to clearing senescent cells prevent, delay, or ameliorate osteoporosis in mouse models. Senolytic compounds are currently being evaluated in interventional clinical trials.

## Conflict of Interest

The authors declare that there is no conflict of interest that could be perceived as prejudicing the impartiality of the research reported.

## Author Roles

All authors contributed to the writing, review, revisions, and approval of the final manuscript.
